# Should Cefoxitin Non-Susceptibility in Ceftriaxone-Susceptible *E. coli* and *K. pneumoniae* Prompt Concerns Regarding Plasmid-Mediated AmpC Resistance? A Genomic Characterization and Summary of Treatment Challenges in Singapore

**DOI:** 10.3390/antibiotics14070722

**Published:** 2025-07-18

**Authors:** Jonathan Jinpeng Foo, Ying Ying Ong, Clement Kin Ming Tsui, David C. Lye, De Partha Pratim, Nurhidayah Binte Mohamed Yazid, Swaine L. Chen, Shawn Vasoo, Tat Ming Ng

**Affiliations:** 1 Department of Pharmacy, Tan Tock Seng Hospital, 11 Jalan Tan Tock Seng, Singapore 308433, Singapore; 2 Infectious Diseases Research Laboratory, National Centre for Infectious Diseases, Tan Tock Seng Hospital, Singapore 308442, Singapore; 3 Lee Kong Chian School of Medicine, Nanyang Technological University, Singapore 308232, Singapore; 4 Department of Infectious Diseases, National Centre for Infectious Diseases, Tan Tock Seng Hospital, Singapore 308442, Singapore; 5 Yong Loo Lin School of Medicine, National University of Singapore, Singapore 117597, Singapore; 6 Department of Laboratory Medicine, Tan Tock Seng Hospital, Singapore 308433, Singapore; 7 Infectious Diseases Translational Research Programme, Department of Medicine, Division of Infectious Diseases, Yong Loo Lin School of Medicine, National University of Singapore, NUHS Tower Block, 1E Kent Ridge Road, Singapore 119228, Singapore; 8 Laboratory of Bacterial Genomics, Genome Institute of Singapore, 60 Biopolis Street, Singapore 138672, Singapore

**Keywords:** cefoxitin non-susceptibility, plasmid-mediated ampC, ampR, DHA-1

## Abstract

**Objectives:** Plasmid-mediated AmpC beta-lactamases represent a growing clinical concern in *Enterobacterales*, with challenges in diagnostic approaches, limited data on clinical outcomes, and our incomplete understanding of their regulatory mechanisms warranting the need for further investigation. **Methods:** This retrospective study examined the genomic and clinical characteristics of cefoxitin-non-susceptible, ceftriaxone-susceptible *Escherichia coli* and *Klebsiella pneumoniae* bloodstream isolates collected from a tertiary hospital in Singapore. Whole-genome sequencing was performed to detect *ampC* genes, subtypes, and associated regulatory elements. **Results:** Among 108 cefoxitin-non-susceptible isolates, only 15 (13.9%) harboured plasmid-mediated *ampC*, suggesting that cefoxitin non-susceptibility alone in ceftriaxone susceptible isolates was not predictive of *ampC* carriage. All plasmid-*ampC* isolates were from the *bla*_DHA-1_ subtype and carried *ampR*, a known transcriptional regulator of inducible beta-lactamase expression. Notably, five non-*ampC* carrying *Klebsiella* isolates displayed truncations in *ompK35* and *ompK36*, which could potentially contribute to reduced cefoxitin susceptibility via porin loss. **Conclusions:** These findings underscore the limited diagnostic utility of cefoxitin susceptibility testing for detecting plasmid-mediated *ampC* producers and highlight the clinical relevance of regulatory genes such as *ampR* in mediating inducible resistance. The routine incorporation of molecular diagnostics or genome sequencing may be necessary to improve detection accuracy and inform antimicrobial stewardship strategies.

## 1. Introduction

AmpC beta-lactamases represent an important cause of antimicrobial resistance among Gram-negative bacteria, posing significant therapeutic and diagnostic challenges in current clinical practice. These Ambler Class C beta-lactamases confer resistance to a broad spectrum of beta-lactam antibiotics including penicillins and extended-spectrum cephalosporins [1]. The primary mechanism underlying this resistance is the hydrolytic opening of the beta-lactam ring via the AmpC enzyme, rendering these antibiotics ineffective [2]. Consequently, clinicians are left with a narrowed repertoire of therapeutic options such as cefepime, carbapenems, or newer beta-lactam/beta-lactamase inhibitor combinations [1]. This reliance on broad-spectrum agents raises multiple concerns regarding antimicrobial stewardship and collateral damage to the microbiome, as well as the acceleration of multidrug-resistant organism emergence [3].

From a clinical perspective, the ability to initiate appropriate and effective antimicrobial therapy in a timely manner is an important determinant of patient outcomes, particularly in more severe infections such as sepsis [4]. Consequently, the timely and accurate identification of AmpC-producing organisms is essential in guiding antimicrobial therapy. While chromosomally encoded AmpC enzymes are well characterized and commonly associated with species such as in *Enterobacter cloacae* complex, *Citrobacter freundii*, and *Serratia marcescens* among others [5], the landscape of AmpC-mediated resistance has evolved significantly over time. Of particular concern is the growing prevalence of plasmid AmpC (pAmpC) enzymes, which can be horizontally transferred into species not inherently associated with AmpCs. However, despite pAmpC enzymes becoming increasingly common in the clinical setting, they frequently go undetected due to limitations in laboratory detection and the absence of established Clinical & Laboratory Standards Institute (CLSI) guidelines [6,7]. This could potentially lead to delays in initiating appropriate antimicrobial therapy and result in suboptimal clinical outcomes.

The accurate detection of pAmpC-harbouring isolates is critical, as these organisms may initially appear susceptible to extended-spectrum cephalosporins. However, treatment with these agents can inadvertently select for resistance through enzyme induction or de-repression, ultimately compromising treatment efficacy [1]. The risk is particularly relevant in species such as *Klebsiella pneumoniae* and *Escherichia coli*, where AmpC production is not typically expected and may be overlooked during empirical treatment selection. In contrast, ceftriaxone-resistant isolates are commonly presumed to be extended-spectrum beta-lactamase (ESBL) producers and, in cases of bacteraemia, are typically treated with carbapenems, which are also effective against AmpC producers [8]. Thus, the greater clinical concern lies in the lack of recognition of pAmpC in ceftriaxone-susceptible isolates, where reliance on extended-spectrum cephalosporins may drive resistance and negatively impact clinical outcomes.

Traditionally, cefoxitin non-susceptibility has been proposed as a screening method for AmpC detection, but this approach carries several limitations [9]. Nonetheless, some clinicians still consider it a reasonable proxy, and it is often suggested as a surrogate marker despite limited supporting evidence, particularly in Southeast Asia [10]. Molecular detection methods are also not routinely incorporated into clinical practice. This is particularly crucial in ceftriaxone-susceptible isolates, in which the use of extended-spectrum beta-lactams should be avoided in these cases.

To address these gaps, our study aimed to investigate the prevalence of *ampC* genes and subtypes and the associated regulatory elements in cefoxitin-non-susceptible *E. coli* and *K. pneumoniae* bacteraemia, as well as to evaluate their treatment outcomes. In addition, other resistance mechanisms associated with beta-lactam resistance were also characterized using a whole-genome sequencing approach to provide a more comprehensive picture to inform therapeutic decisions regarding the choice of beta-lactams.

## 2. Results

### 2.1. Antimicrobial Susceptibility Testing and Bioinformatics Analysis

All isolates were susceptible to ceftriaxone, ceftazidime, ertapenem, and meropenem. Among the 108 cefoxitin-non-susceptible isolates (57 *E. coli* and 51 *K. pneumoniae*), 15 (13.9%) harboured the *bla*_DHA-1_ subtype of the *ampC* beta-lactamase gene, consisting of 5 *E. coli* and 10 *K. pneumoniae*. No other *ampC* beta-lactamase genes were detected. A BLAST search of contigs containing *bla*_DHA-1_ in these 15 isolates indicated 99.8–100% identity to plasmids of various *Enterobacterales* species. Among the *ampC*-positive isolates, all *E. coli* isolates possessed *ampD*, *ampG*, and *ampR.* In contrast, this was not observed in all *K. pneumoniae* isolates. Nevertheless, all *K. pneumoniae* isolates possessed at least *ampR*. Additionally, one *K. pneumoniae* isolate harboured *bla*_SHV-27_, an extended-spectrum beta-lactamase gene. The resistance profile of all *ampC*-carrying isolates are detailed in Table 1 below. Among the non-*ampC* isolates, five *K. pneumoniae* isolates exhibited truncations in *ompK35* or *ompK36*, which may have contributed to reduced cefoxitin susceptibility. No porin gene mutations were detected in the *E. coli* isolates. The resistance profiles of the 93 non-*ampC* containing isolates are provided in the Supplementary Materials.

### 2.2. Clinical Characteristics of Patients with Cefoxitin-Non-Susceptible K. pneumoniae and E. coli Isolates

A total of 108 patients had bacteremia with *E. coli* or *K. pneumoniae* isolated that were cefoxitin-non-susceptible yet ceftriaxone susceptible during the study period from 2016 to 2019. The median age of the cohort was 75 years (interquartile range of 64 to 84 years old), and 54.6% were male. The top three sources of infections were urinary (n = 41, 38.0%), hepatobiliary (n = 39, 36.1%), and respiratory infections (n = 14, 13.0%). The majority had community-acquired bacteremia (n = 49, 45.4%), 41 had healthcare-acquired infections (38.0%), and 18 (n = 16.7%) were nosocomial infections. Overall, the 30-day mortality was 14.8% (n = 16). The specific demographics and clinical characteristics of patients with cefoxitin-non-susceptible *E. coli* or *K. pneumoniae* bacteraemia are also further detailed in Table 2.

Overall, there was no statistically significant difference in 30-day all-cause mortality between patients with *DHA-1* and non-*DHA-1* isolates. Similarly, the proportion of patients who received active empiric carbapenem therapy (*p* = 0.450) or active definitive carbapenem therapy (*p* = 0.871) did not differ significantly between the two groups. Among the patients who received active empiric carbapenem treatment (n = 12), there were no deaths within 30 days. In contrast, the 30-day all-cause mortality rate for patients treated with an active empiric non-carbapenem beta-lactam was 16.7% (11 out of 66 patients died), although no statistical difference in mortality rates was detected (*p* = 0.156). Following susceptibility testing, the 30-day all-cause mortality for patients who received active definitive therapy with a carbapenem was 12.5% (3 out of 24 patients died), while for those treated with an active non-carbapenem beta-lactams, the mortality rate was 8.70% (6 out of 69 patients died) (*p* = 0.503).

## 3. Discussion

In our study, only 13.9% of cefoxitin-non-susceptible isolates tested positive for *ampC* genes. We did not observe any mortality differences due to the presence of pAmpC and the use of different active antibiotic classes including carbapenem and non-carbapenem beta-lactams. This finding reinforces existing evidence that cefoxitin non-susceptibility may not be an accurate surrogate marker for pAmpC. Polsfuss et al. [11] similarly reported that only 17.5% of cefoxitin non-susceptible isolates were confirmed to produce AmpC enzymes. This issue is particularly critical in the subgroup of ceftriaxone-susceptible but cefoxitin-non-susceptible isolates, where cefoxitin resistance may lead clinicians to suspect AmpC production and switch to carbapenems. However, our findings suggest that such assumptions should be made with caution. Furthermore, cefoxitin resistance can arise through alternative mechanisms unrelated to AmpC production, including reduced membrane permeability due to porin loss or modification, such as alterations in *ompK35* and *ompK36* in *Klebsiella* spp. [12]. Thus, cefoxitin non-susceptibility alone in ceftriaxone-susceptible isolates may not necessarily warrant the escalation of antibiotic therapy for presumptive AmpC coverage.

All *ampC*-positive isolates in this study were identified as belonging to the *DHA-1* subtype. Additionally, a BLAST search of contigs containing *bla*_DHA-1_ in these isolates indicated 99.8–100% identity to plasmids of various *Enterobacterales* species, suggesting the ongoing circulation and potential horizontal transfer of these plasmids. These findings underscore the need for continued molecular surveillance to monitor the dissemination of plasmid-mediated *DHA-1* locally, particularly those with the potential for silent spread in ceftriaxone-susceptible isolates. In addition, all the *DHA-1*-positive isolates in our study carried an *ampR* gene. AmpR is a LysR-type transcriptional regulator known to modulate AmpC expression in response to beta-lactam exposure, thereby facilitating inducible resistance and contributing to pathogenicity by upregulating capsule synthesis and the modulation of biofilm formation [13]. Based on bioinformatics analysis and the prior literature, we believe that the *ampR* gene was located on the same *bla*_DHA-1_ plasmid in both the *E. coli* and *K. pneumoniae* isolates in our study, consistent with previous reports by Barnaud et al. [14] for *Salmonella Enteritidis*, Fortineau et al. [15] for *K. pneumoniae*, and Giakkoupi et al. [16] for *E. coli*.

The consistent association of *ampR* with DHA-1 is concerning, as it implies that the pathogen could be capable of mediating inducible resistance under beta-lactam exposure. In the context of inducible AmpC beta-lactamases such as DHA-1, the *ampR* gene plays a critical role in mediating transcriptional activation in response to antibiotic pressure [17]. This differs from constitutively expressed AmpC enzymes like CMY-2, which may not require *ampR* for expression [7]. While *E. coli* intrinsically harbours the *ampG* and *ampD* genes, it lacks a chromosomal *ampR*, which explains its inability to induce chromosomal AmpC under normal circumstances [18]. However, if there is an acquisition of *ampR* alongside DHA-1 on a plasmid, this represents a clinically important development as it confers the capacity for inducible resistance independent of the chromosomal context. In contrast, most *Klebsiella* isolates in our study lacked *ampD* and *ampG*, although the functional consequences of this absence remain unclear. Barceló et al. [19] demonstrated that the loss of AmpG reduced DHA-1 induction without markedly impairing bacterial virulence. The extent to which AmpC enzymes can be functionally expressed or regulated in the absence of both *ampD* and *ampG* remains to be elucidated. Further studies are needed to determine the essential regulatory genes required for effective inducible AmpC expression, particularly in *K. pneumoniae*.

This study was limited to a single tertiary care centre in Singapore with a limited sample size, and the antibiotic resistance epidemiology reported may not be easily generalizable. Nevertheless, we believe that our findings are broadly reflective of national trends within Singapore’s healthcare system. Antibiotic susceptibilities should have been re-tested with the determination of minimum inhibitory concentrations; however, we reported susceptibility results directly from blood culture to reflect real-world clinical scenarios. While other *Enterobacterales* species may also harbour pAmpC, they were beyond the scope of our current investigation. Future studies involving larger cohorts and multiple centres are warranted to better characterize the clinical impact of plasmid AmpCs.

## 4. Materials and Methods

A retrospective study was conducted from January 2014 to June 2019 at Tan Tock Seng Hospital, a tertiary care institution with over 2000 beds in Singapore. During this period, *K. pneumoniae* and *E. coli* from blood cultures that were non-susceptible to cefoxitin but susceptible to third-generation cephalosporins were identified through the hospital’s electronic database and retrieved from the microbiology laboratory. Only the first episode of bacteraemia was included for patients with multiple episodes. Patients were excluded if they had polymicrobial bacteraemia, defined as the isolation of two or more pathogens from blood cultures within 48 h of the initial evaluation, regardless of whether the isolates were from the same or different blood cultures. After applying these criteria, 108 isolates were included in the study.

### 4.1. Antimicrobial Susceptibility Testing

Reporting results directly from blood culture susceptibility testing by disc diffusion is part of routine testing in our laboratory. Positive blood cultures (BACTEC FX, Beckton Dickinson, Franklin Lakes, NJ, USA) were processed as follows: Two drops of broth from the positive bottle were added to 3 ml of sterile saline using a venting needle and mixed. The broth was then lawned onto Mueller-Hinton agar plates, and antibiotic discs were added using a disc dispenser. The plates were then incubated at 35 °C in ambient air for 16–18 h, and the zone sizes were interpreted according to the Clinical & Laboratory Standards Institute standards [20].

### 4.2. DNA Extraction and Library Preparation

Archived isolates from the clinical microbiology laboratory were subcultured to blood agar. Genomic DNA was extracted from isolated bacteria using the QIAamp DNA mini kit (Qiagen, Hilden, Germany). DNA samples were quantified using a NanoDrop 2000 (Thermo Scientific, Waltham, MA, USA) and Qubit 2.0 fluorometer (Invitrogen, Carlsbad, CA, USA). The extraction process was performed to ensure that >100 ng/uL of DNA concentration was obtained with a value > 1.8 for the OD 260/280 ratio. The DNA elutes were then stored at 4 °C. Sequencing libraries were prepared using Nextera XT Library Prep Kit (Illumina, San Diego, CA, USA) and sequenced on a HiSeq 4000 (Illumina) using 300 cycles (150 bp paired end) at the Genome Institute of Singapore.

### 4.3. Bioinformatics Analysis

The Illumina raw data were assessed by Fastqc (https://www.bioinformatics.babraham.ac.uk/projects/fastqc/ (accessed on 16 July 2025)), trimmed by Trim Galore v0.6.3 (http://www.bioinformatics.babraham.ac.uk/projects/trim_galore/ (accessed on 16 July 2025)), assembled de novo using SPAdes v.3.9.0 [21] implemented in shovill pipeline (https://github.com/tseemann/shovill (accessed on 16 July 2025)). The assembled genomes were assessed using QUAST v5.0.2 [22]. The sequence type (ST) and antimicrobial resistance genes were predicted from the contigs using multilocus sequence typing (MLST) (https://github.com/tseemann/mlst (accessed on 16 July 2025)) and NCBI databases implemented in ABRicate v0.9, as well as AMRFinderPlus [23], based on >80% coverage and 90% sequence identity. Kleborate v.2 was used to detect the virulence genes, capsule synthesis (K), and lipopolysaccharide (O) loci, as well as resistance genes in *Klebsiella* [24]. To look for the presence/absence of *ampD*, *ampR*, *ampG*, corresponding nucleotide sequences in *E. coli* (NC_000913.3) were retrieved and searched against genome assemblies using BLAST+ [25]. To verify that *bla*_DHA-1_ is a plasmid-mediated enzyme, the *bla*_DHA-1_ bearing contigs of all isolates were searched against NCBI database using BLASTn (https://blast.ncbi.nlm.nih.gov/Blast.cgi?PROGRAM=blastn&BLAST_SPEC=GeoBlast&PAGE_TYPE=BlastSearch (accessed on 16 July 2025)).

### 4.4. Data Collection

Data were collected retrospectively from inpatient charts and included patient demographics, Charlson’s weighted co-morbidity index, empiric and definitive antibiotics, antimicrobial susceptibility of the bacteraemia isolate, and 30-day all-cause mortality. Regarding the place of acquisition, infection was classified as community-acquired, nosocomial (positive blood culture occurring > 2 days of admission), or healthcare-associated (positive blood culture ≤ 2 days of admission, with any of the following risk factors: (i) recent discharge from hospital after ≥ 2 days’ stay, but within 3 months before the bacteraemia episode, (ii) staying at a nursing home or in long-term care facilities, (iii) haemodialysis or received intravenous therapy within 30 days before the bacteraemia episode). The source of bacteraemia was determined by physician diagnosis and compared with US Communicable Disease Centre/National Healthcare Safety Network surveillance definitions for specific infections. Comparisons between groups were made using Mann–Whitney U tests and Chi-squared/Fisher exact tests as appropriate and were performed using IBM SPSS Statistics (Version 29).

## 5. Conclusions

In summary, this study highlights the low proportion of plasmid-mediated AmpCs among cefoxitin-non-susceptible, ceftriaxone-susceptible *E. coli* and *K. pneumoniae* isolates in our setting. This suggests that cefoxitin non-susceptibility might not be a reliable surrogate marker for pAmpC, reinforcing the need for confirmatory molecular testing rather than the presumptive escalation of therapy based on cefoxitin results alone. All *ampC*-positive isolates were of the *DHA-1* subtype and harboured the *ampR* regulatory gene, suggesting potential for inducible resistance and horizontal plasmid transfer. These findings underscore the importance of molecular surveillance to monitor the dissemination of DHA-1 and associated resistance determinants. Further multicentre studies are needed to better understand the clinical implications of pAmpC and to guide optimal antimicrobial stewardship strategies.

## Figures and Tables

**Table 1:** Resistance Profile of the *ampC*-positive isolates with their respective sequence type (ST) and *ampC* gene subtype. The presence of the regulatory genes (*amp D*, *G*, *R*) is also shown, with ‘yes’ denoting the presence of the gene and ‘no’ denoting its absence.

Isolate	Species	ST	*ampC* Gene Subtype	*ampD*	*ampG*	*ampR*	Other Resistance Genes
GASREC26	*E. coli*	95	*DHA-1*	yes	yes	yes	*EC-5*, *TEM-1*
GASREC31	*E. coli*	345	*DHA-1*	yes	yes	yes	*EC-18*, *TEM-1*, *mcr-3*
GASREC40	*E. coli*	38	*DHA-1*	yes	yes	yes	*EC-8*, *TEM-1*
GASREC81	*E. coli*	155	*DHA-1*	yes	yes	yes	*EC-18*
GASREC124	*E. coli*	69	*DHA-1*	yes	yes	yes	*EC-8*
GASREC08	*K. pneumoniae*	4218	*DHA-1*	yes	yes	yes	*OKP-B-2*
GASREC14	*K. pneumoniae*	15	*DHA-1*	no	no	yes	*OXA-1*, *SHV-28*
GASREC18	*K. pneumoniae*	11	*DHA-1*	no	no	yes	*OXA-1*
GASREC21	*K. pneumoniae*	15	*DHA-1*	no	no	yes	*OXA-1*, *SHV-28*
GASREC28	*K. pneumoniae*	611	*DHA-1*	no	no	yes	*SHV-27*
GASREC34	*K. pneumoniae*	37	*DHA-1*	no	no	yes	*OXA-1*, *SHV-11*
GASREC75	*K. pneumoniae*	20	*DHA-1*	no	no	yes	*SHV-187*
GASREC86	*K. pneumoniae*	3415	*DHA-1*	no	no	yes	*SHV-61*, *TEM-1*
GASREC100	*K. pneumoniae*	218	*DHA-1*	no	no	yes	*SHV-1*
GASREC115	*K. pneumoniae*	15	*DHA-1*	no	no	yes	*OXA-1*, *SHV-28*

**Table 2:** Demographics and clinical characteristics of patients with cefoxitin-non-susceptible *E. coli* or *K. pneumoniae* bacteraemia with comparison between patients with *DHA-1* and non-*DHA-1* isolates.

Characteristic, (%) Unless Otherwise Stated	Total (n = 108)	*DHA-1* (n = 15)	Non-*DHA-1* (n = 93)
Age, median (IQR)	75 (64–84)	75 (59.5–84.5)	75 (65–84)
Male	59 (54.6)	6 (40)	53 (57)
Female	49 (45.4)	9 (60)	40 (43)
Chinese	87 (80.6)	12 (80)	75 (80.6)
ICU admission	18 (16.7)	2 (13.3)	16 (17.2)
Charlson’s co-morbidity score, median (IQR)	5 (4–7)	5 (2–7)	5 (4–7)
**Microorganism**			
*Escherichia coli*	57 (52.8)	5 (33.3)	52 (55.9)
*Klebsiella pneumoniae*	51 (47.2)	10 (66.7)	41 (44.1)
**Source of infection**			
Urinary	41 (38)	4 (26.7)	37 (39.8)
Hepatobiliary	39 (36.1)	7 (46.7)	32 (34.4)
Respiratory	14 (13)	1 (6.7)	13 (14)
Intra-abdominal	7 (6.5)	1 (6.7)	6 (6.5)
Intravascular catheter	1 (0.9)	0 (0)	1 (1.1)
Others	6 (5.6)	2 (13.3)	4 (4.3)
**Place of acquisition**			
Nosocomial onset	18 (16.7)	5 (33.3)	13 (14)
Healthcare-associated onset	41 (38)	6 (40)	35 (37.6)
Community-acquired	49 (45.4)	4 (26.7)	45 (48.4)
**Clinical Outcomes**			
30-day all-cause mortality	16 (14.8)	3 (20)	13 (14)
Received active empiric carbapenem	12 (11.1)	2 (13.3)	10 (10.8)
Received active definitive carbapenem	24 (22.2)	3 (20)	21 (22.6)
Received active empiric non-carbapenem beta-lactams	11 (10.2)	3 (20)	8 (8.6)
Received active definitive non-carbapenem beta-lactams	6 (5.6)	2 (13.3)	4 (4.3)
**Active empiric antibiotics used ^a^**			
Amoxicillin/clavulanic acid	22 (20.4)	0 (0)	22 (23.7)
Ceftazidime	1 (0.9)	0 (0)	1 (1.1)
Ceftriaxone	23 (21.3)	1 (6.7)	22 (23.7)
Cefepime	4 (3.7)	0 (0)	4 (4.3)
Levofloxacin	3 (2.8)	0 (0)	3 (3.2)
Ertapenem	1 (0.9)	0 (0)	1 (1.1)
Meropenem	11 (10.2)	2 (13.3)	9 (9.7)
Piperacillin-tazobactam	16 (14.8)	6 (40)	10 (10.8)
**Active definitive antibiotics used ^b^**			
Amikacin	2 (1.9)	0 (0)	2 (2.2)
Ampicillin	1 (0.9)	0 (0)	1 (1.1)
Amoxicillin/clavulanic acid	21 (19.4)	0 (0)	21 (22.6)
Aztreonam	1 (0.9)	0 (0)	1 (1.1)
Sulfamethoxazole-trimethoprim	2 (1.9)	0 (0)	2 (2.2)
Cefazolin	5 (4.6)	0 (0)	5 (5.4)
Ceftriaxone	36 (33.3)	9 (60)	27 (29)
Ceftazidime	1 (0.9)	0 (0)	1 (1.1)
Cefepime	2 (1.9)	0 (0)	2 (2.2)
Ciprofloxacin	2 (1.9)	0 (0)	2 (2.2)
Levofloxacin	2 (1.9)	1 (6.7)	1 (1.1)
Ertapenem	7 (6.5)	2 (13.3)	5 (5.4)
Meropenem	17 (15.7)	1 (6.7)	16 (17.2)
Piperacillin-tazobactam	2 (1.9)	1 (6.7)	1 (1.1)
Not applicable *	6 (5.6)	1 (6.7)	5 (5.4)

^a^ “Active empiric antibiotics” refers to empiric antibiotics that were determined to be susceptible when antimicrobial susceptibility results became available. ^b^ “Active definitive antibiotics” refers to culture-directed therapy with an antimicrobial to which the isolate was susceptible based on antimicrobial susceptibility results. * Not applicable as patient passed on before receiving any definitive antimicrobial therapy.

## Data Availability

The original data presented in the study are openly available at the NCBI website under BioProject accession number PRJ431029 (SAMN47328257–SAMN4738376).

## Supplementary Materials

The following supporting information can be downloaded at: https://www.mdpi.com/article/10.3390/antibiotics14070722/s1, Table S1: Full resistance profile of the non-*ampC E. coli* isolates. Table S2: Full resistance profile of the non-*ampC K. pneumoniae* isolates.

## References

[1] Tamma P.D., Doi Y., Bonomo R.A., Johnson J.K., Simner P.J. (2019). Antibacterial Resistance Leadership Group. A Primer on AmpC β-Lactamases: Necessary Knowledge for an Increasingly Multidrug-resistant World. Clin. Infect. Dis. Off. Publ. Infect. Dis. Soc. Am..

[2] Mora-Ochomogo M., Lohans C.T. (2021). β-Lactam antibiotic targets and resistance mechanisms: From covalent inhibitors to substrates. RSC Med. Chem..

[3] Ahmed S.K., Hussein S., Qurbani K., Ibrahim R.H., Fareeq A., Mahmood K.A., Mohamed M.G. (2024). Antimicrobial resistance: Impacts, challenges, and future prospects. J. Med. Surg. Public Health.

[4] Joo Y.M., Chae M.K., Hwang S.Y., Jin S.-C., Lee T.R., Cha W.C., Jo I.J., Sim M.S., Song K.J., Jeong Y.K. (2014). Impact of timely antibiotic administration on outcomes in patients with severe sepsis and septic shock in the emergency department. Clin. Exp. Emerg. Med..

[5] Meini S., Tascini C., Cei M., Sozio E., Rossolini G.M. (2019). AmpC β-lactamase-producing Enterobacterales: What a clinician should know. Infection.

[6] Philippon A., Arlet G., Jacoby G.A. (2002). Plasmid-Determined AmpC-Type β-Lactamases. Antimicrob. Agents Chemother..

[7] Jacoby G.A. (2009). AmpC beta-lactamases. Clin. Microbiol. Rev..

[8] Tamma P.D., Heil E.L., Justo J.A., Mathers A.J., Satlin M.J. (2024). Infectious Diseases Society of America 2024 Guidance on the Treatment of Antimicrobial-Resistant Gram-Negative Infections. Clin. Infect. Dis..

[9] Hanson N.D. (2003). AmpC β-lactamases: What do we need to know for the future?. J. Antimicrob. Chemother..

[10] Roberts T., Ling C.L., Watthanaworawit W., Cheav C., Sengduangphachanh A., Silisouk J., Hopkins J., Phommasone K., Batty E.M., Turner P. (2024). AmpC β-lactamases detected in Southeast Asian *Escherichia coli* and Klebsiella pneumoniae. JAC-Antimicrob Resist..

[11] Polsfuss S., Bloemberg G.V., Giger J., Meyer V., Böttger E.C., Hombach M. (2011). Practical approach for reliable detection of AmpC beta-lactamase-producing Enterobacteriaceae. J. Clin. Microbiol..

[12] Tsai Y.-K., Fung C.-P., Lin J.-C., Chen J.-H., Chang F.-Y., Chen T.-L., Siu L.-K. (2011). Klebsiella pneumoniae Outer Membrane Porins OmpK35 and OmpK36 Play Roles in both Antimicrobial Resistance and Virulence. Antimicrob. Agents Chemother..

[13] Vadlamani G., Thomas M.D., Patel T.R., Donald L.J., Reeve T.M., Stetefeld J., Standing K.G., Vocadlo D.J., Mark B.L. (2015). The β-Lactamase Gene Regulator AmpR Is a Tetramer That Recognizes and Binds the d-Ala-d-Ala Motif of Its Repressor UDP-N-acetylmuramic Acid (MurNAc)-pentapeptide. J. Biol. Chem..

[14] Barnaud G., Arlet G., Verdet C., Gaillot O., Lagrange P.H., Philippon A. (1998). Salmonella enteritidis: AmpC plasmid-mediated inducible beta-lactamase (DHA-1) with an ampR gene from Morganella morganii. Antimicrob. Agents Chemother..

[15] Fortineau N., Poirel L., Nordmann P. (2001). Plasmid-mediated and inducible cephalosporinase DHA-2 from Klebsiella pneumoniae. J. Antimicrob. Chemother..

[16] Giakkoupi P., Tambic-Andrasevic A., Vourli S., Skrlin J., Sestan-Crnek S., Tzouvelekis L.S., Vatopoulos A.C. (2006). Transferable DHA-1 cephalosporinase in Escherichia coli. Int. J. Antimicrob. Agents..

[17] Akata K., Muratani T., Yatera K., Naito K., Noguchi S., Yamasaki K., Kawanami T., Kido T., Mukae H. (2019). Induction of plasmid-mediated AmpC β-lactamase DHA-1 by piperacillin/tazobactam and other β-lactams in Enterobacteriaceae. PLoS ONE.

[18] Honoré N., Nicolas M.H., Cole S.T. (1986). Inducible cephalosporinase production in clinical isolates of Enterobacter cloacae is controlled by a regulatory gene that has been deleted from *Escherichia coli*. EMBO J..

[19] Barceló I.M., Escobar-Salom M., Cabot G., Perelló-Bauzà P., Jordana-Lluch E., Taltavull B., Torrens G., Rojo-Molinero E., Zamorano L., Pérez A. (2024). Transferable AmpCs in Klebsiella pneumoniae: Interplay with peptidoglycan recycling, mechanisms of hyperproduction, and virulence implications. Antimicrob. Agents Chemother..

[20] (2025). Performance Standards for Antimicrobial Susceptibility Testing.

[21] Bankevich A., Nurk S., Antipov D., Gurevich A.A., Dvorkin M., Kulikov A.S., Lesin V.M., Nikolenko S.I., Pham S., Prjibelski A.D. (2012). SPAdes: A new genome assembly algorithm and its applications to single-cell sequencing. J. Comput. Biol. J. Comput. Mol. Cell Biol..

[22] Gurevich A., Saveliev V., Vyahhi N., Tesler G. (2013). QUAST: Quality assessment tool for genome assemblies. Bioinforma Oxf. Engl..

[23] Feldgarden M., Brover V., Haft D.H., Prasad A.B., Slotta D.J., Tolstoy I., Tyson G.H., Zhao S., Hsu C.H. (2019). Validating the AMRFinder Tool and Resistance Gene Database by Using Antimicrobial Resistance Genotype-Phenotype Correlations in a Collection of Isolates. Antimicrob. Agents Chemother..

[24] Wyres K.L., Nguyen T.N.T., Lam M.M.C., Judd L.M., Chau Nvan V., Dance D.A.B., Ip M., Karkey A., Ling C.L., Miliya T. (2020). Genomic surveillance for hypervirulence and multi-drug resistance in invasive Klebsiella pneumoniae from South and Southeast Asia. Genome Med..

[25] Camacho C., Coulouris G., Avagyan V., Ma N., Papadopoulos J., Bealer K., Madden T.L. (2009). BLAST+: Architecture and applications. BMC Bioinform..

