# Supplementary material for: Should Cefoxitin Non-Susceptibility in Ceftriaxone-Susceptible E. coli and K. pneumoniae Prompt Concerns Regarding Plasmid-Mediated AmpC Resistance? A Genomic Characterization and Summary of Treatment Challenges in Singapore
Source: Antibiotics (Basel). 2025 Jul 18;14(7):722. doi: 10.3390/antibiotics14070722 (PMC12291829; doi:10.3390/antibiotics14070722)
Supplement: Supplementary file 1 [file antibiotics-14-00722-s001.zip › antibiotics-3730877-supplementary.pdf]

Supplementary Materials

Table S1: Full resistance profile of the non-*ampC E. coli* isolates

Isolate #	ST	*bla*$_{OXA-1}$	*bla*$_{EC}$ (chromosomal)	*bla*$_{TEM}$	*mcr*
GASREC 01	226		EC		mcr-1
GASREC 02	38		EC-8	TEM-1	
GASREC 05	38		EC-8	TEM-1	
GASREC 09	224		EC-18	TEM-1	
GASREC 10	-		EC-18		
GASREC 12	569		EC-5		
GASREC 13	405		EC-8		
GASREC 15	38		EC-8		
GASREC 19	405	OXA-1	EC-8		
GASREC 20	359	OXA-1	EC-8		
GASREC 22	457		EC-8	TEM-1	
GASREC 23	131		EC-5	TEM-1	
GASREC 24	12629		EC-8		
GASREC 25	405		EC-8		
GASREC 29	-		EC-18	TEM-1	
GASREC 30	131		EC-5		
GASREC 33	1670		EC		
GASREC 36	1196		EC-18	TEM-1	mcr-1
GASREC 38	38		EC-8		
GASREC 45	38		EC-8		
GASREC 46	457		EC-8		
GASREC 47	-		EC-15		
GASREC 50	8492		EC-18		
GASREC 51	-		EC-8	TEM-1	
GASREC 53	155		EC-18	TEM-1	mcr-1
GASREC 54	131		EC-5	TEM-1	
GASREC 55	405		EC-8	TEM-1	
GASREC 56	162		EC-18	TEM-1	
GASREC 59	-		EC-18		
GASREC 61	155		EC-18		
GASREC 63	410		EC-15		

GASREC 64	1722		EC-19	
GASREC 65	-		EC-18	TEM-1
GASREC 66	48		EC-15	
GASREC 67	648		EC-19	
GASREC 68	38		EC-8	TEM-1
GASREC 69	95		EC-5	TEM-1
GASREC 70	38		EC-8	TEM-135
GASREC 73	135		EC-5	
GASREC 76	354		EC-19	
GASREC 77	38		EC-8	
GASREC 83	131		EC-5	TEM-1
GASREC 84	405		EC-8	
GASREC 85	1193		EC-5	TEM-1
GASREC 104	69		EC-8	
GASREC 105	38		EC-8	
GASREC 109	131	OXA-1	EC-5	
GASREC 113	73		EC-5	
GASREC 114	131		EC-5	
GASREC 126	-		EC-13	
GASREC 128	648		EC-19	
GASREC 129	156		EC-18	

Supplementary Materials

Table S2: Full resistance profile of the non-*ampC K. pneumoniae* isolates

Isolate #	ST	*bla*OXA-1	*bla*SHV	*bla*TEM	*omp* porin mutations
GASREC 03	65				
GASREC 04	231		SHV-1		OmpK36
GASREC 06	273	OXA-1	SHV-11		
GASREC 11	15		SHV-28		
GASREC 16	-				
GASREC 17	86		SHV-1		
GASREC 27	65		SHV-11		
GASREC 32	33	OXA-1	SHV-108		
GASREC 35	1010		SHV-11		
GASREC 37	37		SHV-11		
GASREC 41	23		SHV-11		
GASREC 43	515		SHV-11		
GASREC 44	15		SHV-28		
GASREC 48	138				
GASREC 52	502		SHV-62		
GASREC 57	-		SHV-108		
GASREC 58	147		SHV-11	TEM-1	
GASREC 60	60		SHV-11		
GASREC 62	485		SHV-52		
GASREC 71	348		SHV-11		
GASREC 72	-		SHV-11		
GASREC 78	1102		SHV-11		
GASREC 80	2607				
GASREC 82	37		SHV-11		OmpK36
GASREC 87	29		SHV-187		
GASREC 88	562		SHV-85		
GASREC 90	1630		SHV-1		
GASREC 91	86		SHV-1		
GASREC 93	231		SHV-1		
GASREC 94	-		SHV-11		
GASREC 98	268		SHV-203		

Supplementary Materials

GASREC 99	111	SHV-11	TEM-1	
GASREC 103	23	SHV-11		
GASREC 110	592	SHV-26		
GASREC 111	23	SHV-11		
GASREC 112	2503			
GASREC 116	23	SHV-11		OmpK35
GASREC 117	592	SHV-26		
GASREC 123	-	SHV-1		OmpK36
GASREC 125	23	SHV-11		
GASREC 127	1602			OmpK35, OmpK36
